# Characteristics and treatments for pediatric ordinary and incarcerated inguinal hernia based on gender: 12-year experiences from a single center

**DOI:** 10.1186/s12893-020-01039-5

**Published:** 2021-02-01

**Authors:** Kai Wang, Sarah Siyin Tan, Yue Xiao, Zengmeng Wang, Chunhui Peng, Wenbo Pang, Dongyang Wu, Yajun Chen

**Affiliations:** Department of General Surgery, Beijing Children’s Hospital, Capital Medical University, National Center for Children’s Health, No.56 Nanlishi St, Xicheng District, Beijing, 100045 China

**Keywords:** Pediatric, Inguinal hernia, Hernia repair, Incarcerated hernia

## Abstract

**Background:**

Congenital primary inguinal hernia is a common condition among children. Although much literature regarding inguinal hernia is available, large scale analysis are few, and rarely do they expand on gender difference or incarcerated hernias.

**Methods:**

Patients with unilateral or bilateral inguinal hernia who were admitted to our hospital and received open inguinal hernia repair (OIHR) or laparoscopic inguinal hernia repair (LIHR) under general anesthesia were included. LIHR was performed using single-site laparoscopic percutaneous extraperitoneal closure (SLPEC). Medical records were retrospectively collected and analyzed.

**Results:**

A total of 12,190 patients were included in this study. The ratio of male to female was 4.8:1. There was a total of 10,646 unilateral hernias (87.3%) and 1544 bilateral hernias (12.7%), with a corresponding ratio of 6.9:1. 12,444 hernia repair surgeries, 11,083 (89.1%) OIHR and 1361 (10.9%) LIHR, were held. OIHR had a shorter operative time than LIHR for all unilateral and female bilateral repair, unlike for bilateral male repair. There was no difference between OIHR and LIHR for ipsilateral recurrent hernia in males. There was a difference between OIHR and LIHR for metachronous contralateral hernia. Incarcerated inguinal hernia was associated with longer operative time, hospital stay and higher hospital costs. Females and patients under 1 year were more likely to present with incarcerated hernia.

**Conclusions:**

OIHR should be considered for male patients, especially for unilateral and complete inguinal hernia. LIHR is highly recommended for female patients. For incarcerated hernia, attention should be paid to patients under 1 year old, as they can be 60 times more susceptible, and females. Surgeons should also be aware of ovary hernias in females.

## Background

Congenital primary inguinal hernia is a common condition among children and with an estimated 20 million cases worldwide each year, hernia repair is postured to be the most frequent surgical procedure within the pediatric population [1, 2]. Inguinal hernia affects 0.8–4.4% of all children [3], with higher incidence rates among males, preterm infants and infants with lower birth weights [1, 4–7].

Although much literature regarding inguinal hernia is available, large scale analysis are few, and rarely do they expand on gender difference or incarcerated hernias. Thus, our goal was to describe the different characteristics of pediatric inguinal hernia in a large population for both genders based on our 12-year experience, and to accordingly recommend one of the two commonly used methods of hernia repair: the traditional open inguinal hernia repair (OIHR) and laparoscopic inguinal hernia repair (LIHR), conducted by single-site laparoscopic percutaneous extraperitoneal closure (SLPEC) [8]. An additional goal was to describe the characteristics of incarcerated inguinal hernia.

## Methods

### Patients

A total of 12,190 patients who were admitted to the Department of General Surgery in Beijing Children’s Hospital diagnosed as inguinal hernia during the span of 2007 to 2019 were included in this retrospective study. A history of inguinal reversible mass, with positive physical examination (demonstrable hernia or incarcerated hernia) and inguinal region ultrasound confirmation was used as the criteria for diagnosis. Children who received conservative treatment were removed from the study, and children who received hernia repair under general anesthesia were included. Follow up in the outpatient clinic was held. Retrospective collection of all patients’ data was collected from medical records.

### Operation approach

Hernia repair was carried out via high ligation of the hernia sac and performed as either OIHR or LIHR. Before the year 2012, all patients received OIHR. The incision was chosen at the surface projection of the external inguinal ring, and the inguinal ligament was not opened. After individually dragging and separating the layers outside the outer inguinal ring, the hernia sac was ligated at the height of the peritoneum and totally transected.

With the growing demand of parents and the improvement of anesthesia, our hospital introduced LIHR in 2012. LIHR was adopted for male patients who were older than 1 year of age, who had no other disease that could potentially affect anesthesia, who presented with inguinal hernia that was not very large, and whose parents requested for laparoscope surgery. OIHR remained the surgery of choice for other male patients. For female patients, LIHR was always recommended unless the patient had another disease that could affect anesthesia or if her parents strongly preferred OIHR. LIHR was performed using single-site laparoscopic percutaneous extraperitoneal closure (SLPEC). A single 5 mm trocar was placed at the umbilical region to create pneumoperitoneum and to insert a 5 mm laparoscope. A double suture hernia needle was inserted at the surface projection of the internal inguinal ring, and the peritoneum was punctured after threading a half circle around the hernia sac. The needle was then returned to the starting point and the remainder of the circle was threaded. Lastly, the needle was penetrated through the abdominal wall at the site of the original puncture hole, securing the suture and closing the hernia sac via the extraperitoneal knot. Contralateral exploration was performed and all contralateral patent processus vaginalis (PPV) were repaired simultaneously for the potential contralateral synchronous hernia (CSH) during LIHR.

### Statistical analysis

All the data were analyzed using SPSS for Windows version 17.0. Normal distribution data was presented by (mean ± standard deviation). Non-normal distribution data was presented by median [interquartile range (IQR) first quartile–third quartile]. Categorical variables were presented by frequencies and percentages. Chi-square test and Mann Whitney U test was used to establish significance among two and single categorical data groups respectively, *P* < 0.05 was considered statistically significant.

## Results

A total of 12,190 patients, 10,072 male (82.6%) and 2118 female (17.4%), were included in this study. The ratio of male to female was 4.8:1. The median age of patients at time of surgery was 3 years 1 month (IQR 1 year 8 months–5 years 6 months) and ranged from 1 day to 17 years 8 months. There was a total of 10,646 unilateral hernias (87.3%) and 1544 bilateral hernias (12.7%), with a corresponding ratio of 6.9:1. The ratio of right: left: bilateral hernia was 4.1:2.8:1 (6372:4274:1544). The incidence of bilateral hernia in male and female patients was 8.2% and 4.4% respectively, with a corresponding ratio of 1.9:1. Demographics of the patients were described in Table 1.

**Table 1:** Demographics of patients with inguinal hernia established during hernia repair operation

Variables	OIHR (N = 11,083)		LIHR (N = 1361)	
	Male	Female	Male	Female
Right	5394	605	278	226
Left	3470	562	160	204
Bilateral	822	230	184	309
Total	9686	1397	622	739

*OIHR* open inguinal hernia repair, *LIHR* laparoscopic inguinal hernia repair

**Fig. 1:**
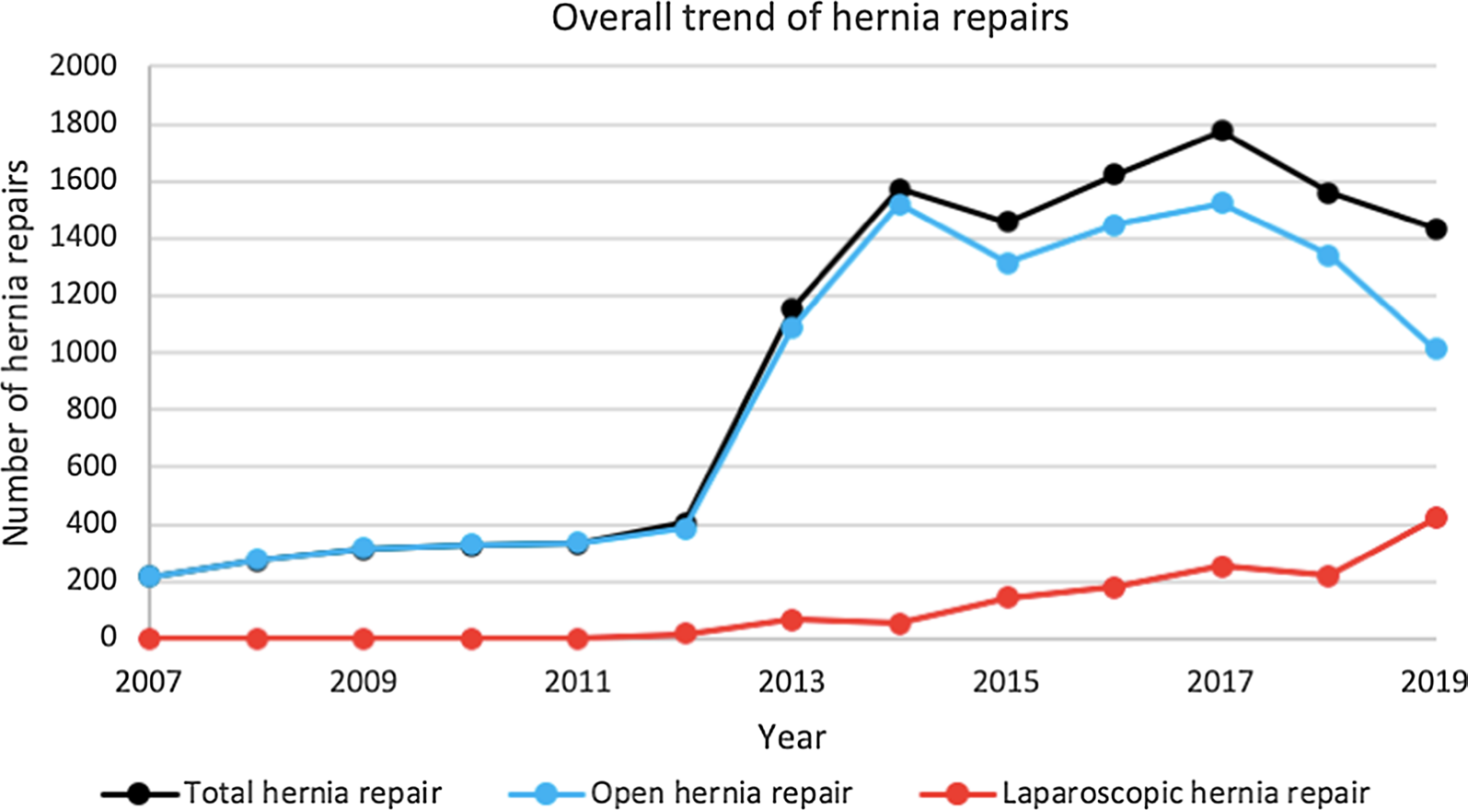
Overall trend of hernia repairs

**Fig. 2:**
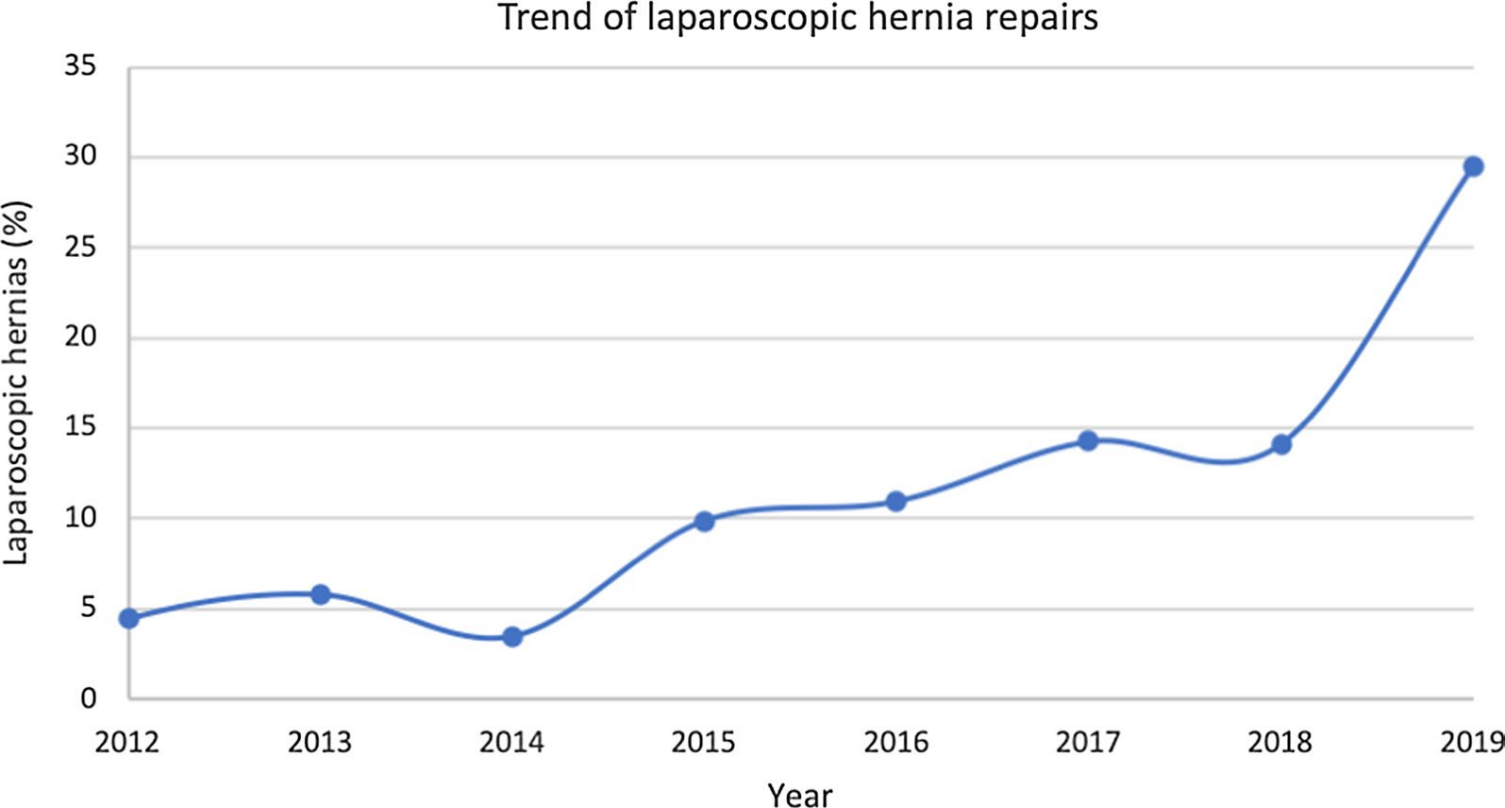
Trend of laparoscopic hernia repairs

The included 12,190 patients underwent a total of 12,444 hernia repair surgeries (235 males and 18 females received 2 surgeries, 1 male received 3 surgeries, the rest received only 1 surgery). Of the 12,444 surgeries, 11,083 (89.1%) were OIHR and 1361 (10.9%) were LIHR. There was an obvious increase in hernia repair surgeries over the years, with only 216 surgeries in 2007 and 1433 surgeries in 2019 (Fig. 1). The greatest increase per-year was from 2012 to 2013, where the number of surgeries almost tripled from 405 to 1155, and the year 2012 was also the first year that we began LIHR. The increase was not linear, with the most surgeries in 2017 and a slight drop in the following two years. Although not linear, there was also a general increase of LIHR throughout the years, 18 in 2012 to 422 in 2019 (Fig. 2).

The operative time was 15 (IQR 10–22) minutes and 31 (IQR 25–40) minutes for unilateral and bilateral hernia repair respectively. For unilateral hernia repair, OIHR had a significantly shorter operative time than LIHR for both male and female patients (both *P* < 0.01). For female patients undergoing bilateral hernia repair, OIHR also had a significantly shorter operative time than LIHR (*P* = 0.01). There was no statistical difference for operative time in male patients undergoing bilateral hernia repair (P = 0.25). Table 2 provides further details.

**Table 2:** Comparisons between OIHR and LIHR for non-incarcerated hernia

Variables	OIHR	LIHR	Result (Z/χ^2^)	*P*
Operative time (Min)				
Male				
Unilateral	15 (IQR 10–20)	30 (IQR 23–39)	22.02	< 0.01
Bilateral	33 (IQR 24–46)	35 (IQR 30–41)	1.15	0.25
Female				
Unilateral	15 (IQR 10–20)	24 (IQR 20–32)	16.35	< 0.01
Bilateral	24 (IQR 20–30)	30 (IQR 24–36)	2.59	0.01
Ipsilateral recurrent hernia				
Male				
Yes	36	3	0.20	0.66
No	9650	619		
Female				
Yes	0	3	5.68	0.02
No	1397	736		
Metachronous contralateral hernia				
Male				
Yes	195	1	10.75	< 0.01
No	9491	621		
Female				
Yes	15	1	5.73	0.02
No	1382	733		

Chi-square test and Mann Whitney U test was used to establish significance among two and single categorical data groups, and results were displayed using Z and χ^2^ respectively

*OIHR* Open inguinal hernia repair, *LIHR* Laparoscopic inguinal hernia repair, *Min* minutes

P < 0.05 was considered statistically significant

There was a total of 42 patients who received surgery for ipsilateral recurrent hernia (IRH). 36 (85.7%) and 6 (14.3%) had respectively received OIHR and LIHR as their initial hernia repair. Chi-square test did not reveal a statistically significant difference between OIHR and LIHR for IRH in male patients (*P* = 0.66) but revealed a significant difference in female patients (*P* = 0.02). Hernia repair for IRH was carried out between 81 days and 4 years 4 months after the initial repair (median, 11 months 22 days).

212 patients received hernia repair for metachronous contralateral hernia (MCH) at a later date. 210 (99.1%) and 2 (0.9%) had respectively received OIHR and LIHR as their initial hernia repair. Chi-square test revealed a statistically significant difference between OIHR and LIHR for MCH in male and female patients (*P* < 0.01, *P* = 0.02 respectively). Hernia repair for MCH was carried out between 7 days to 7 years 5 months after the initial repair (median, 9 months 19 days).

Apart from the ordinary hernia, 69 patients, including 49 (71.0%) males and 20 (29%) females, underwent emergent hernia repair due to incarcerated hernia. Of these patients, 61 (88.4%) and 8 (11.6%) respectively received OIHR and LIHR. Hernia contents are as follows: 47 (68.1%) intestine, 19 (27.5%) ovary, 2 (2.9%) appendix and 2 (2.9%) omentum. Of the incarcerated intestines, 2 (4.3%) presented with intestinal obstruction and perforation respectively, and 1 (2.1%) presented with meckel's diverticulum and intestinal necrosis respectively. The hernia content of 1 child with a perforated intestine included the appendix. Of the incarcerated ovaries, 3 (15.8%) included the fallopian tube, of which 1 also partly included the fundus of the uterus. In comparison with unilateral non-incarcerated hernia, patients with incarcerated hernia had a significantly longer operative time, longer hospital stay and higher hospital cost (all *P* < 0.01). Furthermore, chi-square test revealed a statistically significant difference for both gender and age (both *P* < 0.01). Male and female patients had a respective incarcerated rate of 0.52% and 1.25%, and a 1:2.40 ratio, which inferred a female prevalence.

Patients under the age of 1 year had an incidence of 13.28% for incarcerated hernia, which decreased sharply to 0.21% in patients over the age 1, the ratio was 63.2:1. Table 3 provides further details.

**Table 3:** Comparisons between incarcerated hernia and non-incarcerated hernia

Variables	Incarcerated hernia	Non-incarcerated hernia (unilateral)	Result (Z/χ^2^)	*P*
Gender				
Male	49	9259	11.37	< 0.01
Female	20	1580		
Age (year)				
≤ 1	47	307	930.62	< 0.01
> 1	22	10,532		
Operative time (Min)	57 (IQR 36–73)	15 (IQR 10–22)	7.16	< 0.01
Hospital length (days, median)	4	1	50.90	< 0.01
Hospital Cost (RMB)	7106.89 (IQR 5476.00–10,135.08)	2484.58 (IQR 2088.66–2949.37)	12.12	< 0.01

Chi-square test and Mann Whitney U test was used to establish significance among two and single categorical data groups, and results were displayed using Z and χ^2^ respectively

*Min* minutes, *RMB* Renminbi, Chinese Yuan

P < 0.05 was considered statistically significant

## Discussion

Inguinal hernia is a common disease in pediatric patients with an estimated incidence of 1–5% [1] and hernia repair compromises of up to 15% of all operations in some pediatric centers [9]. Since the 1960s, Beijing Children’s Hospital has implemented several measures to meet the increasing demands of inguinal hernia patients in the outpatient department [10], which we attribute to heightened awareness of the disease in parents (Fig. 1). The concept of day surgery has gradually gained international recognition and is now acknowledged as a practice which benefits patients with timely surgery. Reviewing our 12-year experience of 12,190 patients between 2007 to 2019, we have summarized the characteristics and treatment results for pediatric ordinary and incarcerated inguinal hernia based on gender, and provide our recommendations accordingly. To our knowledge, this study boasts the largest number of pediatric inguinal hernia participants.

Based on the results, we concluded that the ratio of male to female inguinal hernia was 4.8:1, which was higher than the reported 2.5:1 [9]. Our study boasts of participant volume almost 10–12 times more than the reported articles [9] and we postulate that our result may be a better representation of the true morbidity. Additionally, the ratio of unilateral: bilateral and right: left: bilateral was 6.9:1 and 4.1:2.8:1 respectively, equal to other studies [9].

OIHR is the conventional surgical approach for the treatment of pediatric inguinal hernia and has been extensively adopted for years, whereas LIHR was introduced due to the rising demand for minimally invasive procedures and smaller incisions. Since its introduction, the choice between OIHR and LIHR has always been a controversial issue. Some studies have recommended LIHR for its aesthetical benefits and ability to repair CSH simultaneously. One distinct difference is that SPLEC, our method for LIHR, does not require laparoscopic suture skills [8]. Because proper suturing of the PPV is fundamental for successful hernia repair, the opportunity to sidestep laparoscopic suture therefore also potentially sidesteps re-surgery. Nonetheless, no study comparing OIHR and LIHR has been concerned with the differences between male and female pediatric patients. In this study, we analyzed the relevant data and addressed these differences.

Among males, OIHR had a shorter operative time for unilateral hernias (*P* < 0.01), and no difference for bilateral hernias (*P* = 0.25). At a median time of only 15 min, operative time for OIHR was about half that of LIHR, and this was also much shorter than other reported operative times [3]. This may be attributed to two factors. One, OIHR is a very common operation in our department. As such, the operative time was already appreciably short, due to both the continuous refinement of operative skills spanning nearly 60 years as well as the accompaniment of safe and effective anesthesia. Two, LIHR was only introduced in 2012, and the surgeons had less than 10 years of experience which resulted in a relatively longer operative time. There was no difference between OIHR and LIHR in male patients for ipsilateral recurrence (*P* = 0.66) but results showed that MCH was lower in LIHR (*P* < 0.01). Based on this result, we suggest that centers with vast experience in OIHR should consider OIHR for male patients as a shorter operative time would be more beneficial. Nonetheless, surgeons should be vigilant about the possibility of MCH.

Among female patients, results showed that OIHR had shorter operative time for both unilateral and bilateral hernia (*P* < 0.01, *P* = 0.01). When making comparisons with OIHR, other centers have reported a shorter LIHR operative time for both unilateral and bilateral hernia [11], and we agree that the operative time could be shorter with more experience. Although LIHR had a lower MCH rate than OIHR (*P* = 0.02), it also had a higher IRH rate (*P* = 0.02). Reviewing the 3 IRH female patients, we found that they all received surgery in 2013, which was only a year after the introduction of LIHR in our center. No other recurrence was found. We attribute the 3 IRH to unfamiliarity with LIHR and nonetheless recommend that female patients receive LIHR. It should be emphasized that the surgeon’s learning curve is substantial with LIHR [12]. When a surgeon is first introduced to the surgery, confirmation that ligation of the hernia sac is crucial to avoid potential IRH, regardless of additional operative time. Once surgeons become well acquainted with the surgical demands of LIHR, it’s use among female patients could be highly beneficial.

Firstly, compared to male patients, LIHR is safer for female patients. In male patients, frequency of thin and weak hernia sacs is higher, and there were even a number of complete inguinal hernia whereby the PPV was entirely open. These patients generally presented with a giant reversible inguinal mass along with an extremely thin hernia sac that was particularly prone to tear during surgery. We also witnessed several cases where due to the thin hernia sac, the vas deferens seemed to be inside the hernia. It was very difficult to integrally dissociate the thin hernia sac with OIHR, thus LIHR would not have been appropriate for this group of patients. Secondly, the spermatic cord and vas deferens, anatomical structures unique to males, is close to the posterior wall of the hernia sac, which increases the difficulty of dissection and ligation. Once injured, it could lead to severe complications. However, for female patients, the round ligament of the uterus is located at the posterior wall of the hernia sac, allowing for easier and safer dissection and ligation during LIHR [13]. Thirdly, female patients tend to be more concerned with aesthetics, thereby rendering LIHR as a preferred choice. Fourthly, LIHR provides the ability for exploration of uterus and ovaries, which is of great significance for future reproduction. Should any problems be identified, relevant surgery can also be given immediately. Fifthly, LHIR does not require extensive dissection of surrounding tissues and causes less disturbance to the normal anatomy [14]. Last but not least, the total incidence of bilateral hernia in female patients is higher. This study found that female patients presented with bilateral hernia 1.9 times more frequently than male patients. Contralateral exploration for PPV and simultaneous repair for the potential CSH can be easily carried out with LHIR, reducing potential emotional and financial burden for the family [15]. Additionally, since SPLEC does not require laparoscopic suturing of the PPV, when done accurately, it will also ensure a lower recurrence rate [8]. This study therefore highly recommends LIHR for female patients.

Different from the ordinary hernia, results showed that operative time for incarcerated hernia was nearly 4 times longer, total hospital financial cost was 2.9 times higher, and hospitalization length was 4 times longer. When comparing the demographics data, we found that female patients had higher incidence, which was about 2.4 times greater than male patients. This can be explained by the fact that contents of incarceration for male patients only included the intestine or colon and thus bimanual reduction was sufficient. However for females, the ovary was the most common incarcerated hernia content besides the intestine. This could be related to the presence of a short round ligament [16]. Assessment of blood supply for an incarcerated ovary can sometimes be difficult and bimanual reduction tends to be more challenging, which explains why female patients have a higher chance of requiring emergent surgery. In addition, there were fewer female patients than male patients, which also contributed to the discrepancy in incidence between the two genders.

Another finding was that patients under the age of 1 year were more than 60 times more susceptible to incarcerated hernias than patients older than 1 year old. Explanation for this result can be attributed to the following 3 aspects. Firstly, PPV has a tendency of narrowing or obliterating as patients grow older [17], which in turn decreases the incidence of incarcerating. Secondly, patients under the age of 1 year old are unable to communicate either the telltale presence of an inguinal hernia or the pain and discomfort that might be associated with it. Inguinal hernia might only be noticed by caregivers when incarcerated. Thirdly, some parents become more aware of the possibility of a hernia incarcerating and can call for bimanual reduction before it happens. We hence conclude that more attention should be given to male patients under 1 year old in order to reduce the incidence of severe incarcerated hernias. Observation for incarcerated ovarian hernia in females should also be emphasized.

This study has some limitations. Firstly, this was a retrospective study and we did not randomize the selection of surgical repair, thereby causing selection bias, which might have indirectly caused a predisposition of success toward LIHR for females. Randomized selection for OIHR and LIHR would further indorse our findings. Secondly, we did not carry out an extensive postoperative long-term follow up, and potentially missed out on information regarding post-surgical complications such as hematoma and wound infection, as well as the issue of long-term fertility function. However, should the PPV have been inadequately obliterated, it would have presented itself shortly after operation, during the post-operation follow up in our clinic, and thus would have been reflected in our study. Nonetheless, extensive follow-up would still be beneficial in providing additional reliability.

## Conclusions

From this large-scale single center retrospective study, we concluded that OIHR should be considered for male patients, especially when dealing with unilateral hernia and complete inguinal hernia. In these cases, surgeons should be vigilant about the possibility of MCH. Additionally, LIHR is highly recommended for female patients. To reduce the incidence of severe incarcerated hernias, special attention should be paid to patients under 1 year old, as they can be up to 60 times more susceptible, and female patients. Surgeons should also be aware of ovary hernias in females.

## Data Availability

Data generated or analyzed during this study are included in this published article (Tables 1, 2, 3 and Figs. 1, 2). Additional raw data is available from the corresponding author upon reasonable request.

## Abbreviations

OIHR: Open inguinal hernia repair
LIHR: Laparoscopic inguinal hernia repair
SLPEC: Single-site laparoscopic percutaneous extraperitoneal closure
PPV: Patent processus vaginalis
CSH: Contralateral synchronous hernia
IQR: Interquartile range
IRH: Ipsilateral recurrent hernia
MCH: Metachronous contralateral hernia
